# The reliability and validity of decreased sound tolerance scale-screening

**DOI:** 10.1016/j.bjorl.2021.11.009

**Published:** 2021-12-23

**Authors:** Serpil Allusoglu, Songul Aksoy

**Affiliations:** a Izmir Bakircay University, Faculty of Health Sciences, Department of Audiology, Izmir, Turkey; b Hacettepe University, Faculty of Health Sciences, Department of Audiology, Ankara, Turkey

**Keywords:** Hyperacusis, Scan, Scale

## Abstract

**Objectives:**

Decreased Sound Tolerance (DST) is a negative reaction to a sound that does not cause any reaction in an individual with normal hearing. DST’s subclasses include hyperacusis, phonophobia, and misophonia, which are distinct and have therapy variations. There is no diagnostic method or scale that distinguishes them in the literature. This study’s purpose was to develop a screening scale that distinguishes these three DSTs.

**Methods:**

The study comprised 257 willing participants with normal hearing. Cronbach alpha coefficient, item-total correlation, and item differentiation of the Decreased Sound Tolerance Scale-Screening (DSTS-S) were evaluated. Structural validity of DSTS-S was performed by Varimax rotation with Kaiser normalization using Explanatory Factor Analysis (EFA), and Confirmatory Factor Analysis (CFA) was performed to assess its structural compatibility. The scale’s total scores from each section were compared using the Mann-Whitney *U* test in symptom (+) and symptom (–) participants.

**Results:**

The Cronbach alpha value for hyperacusis, phonophobia, and misophonia sections of DSTS-S was calculated as 0.881, 0.775, and 0.938, respectively. The difference between the independent sample*t*-test and the variables was statistically significant (p < 0.01). The Mann-Whitney *U* test showed a significant difference between the median values of the total groups’ scores with and without hyperacusis, phonophobia, and misophonia (HTS, PTS, and MTS, respectively) (p < 0.05). Evaluation by ROC analysis showed that hyperacusis was useful in predicting the presence of hyperacusis, phonophobia was useful in predicting the presence of phonophobia, and misophonia was useful in predicting the presence of misophonia (*p* < 0.001). Hyperacusis and misophonia sections showed high reliability, and phonophobia section showed a moderately reliable level. The Kappa test showed that the compatibility between test–retest for the total scores was statistically significant (*p* < 0.001).

**Conclusion:**

The study’s results indicated that DSTS-S is a valid and reliable scale for identifying subtypes/problems/classes of decreased sound tolerance.

## Highlights

• Decreased Sound Tolerance Scale-Screening is easy to apply and takes a short time.
• This scale distinguishes hyperacusis, phonophobia, misophonia.
• This scale guides clinician to which discipline the patient should be referred.

## Introduction

Different terms have been used for decades to express Decreased Sound Tolerance (DST), which is defined as a negative reaction to a sound that does not cause any reaction in an average person. The most commonly used term to describe hypersensitivity to sounds is hyperacusis, whereas DST involves multiple phenomena. Jastreboff and Jastreboff divided DST into three subclasses: hyperacusis, misophonia, and phonophobia. These three subclasses are different DSTs that occur as a result of different processes.^1^, ^2^, ^3^, ^4^, ^5^

From a behavioral perspective, an individual with hyperacusis experiences extreme discomfort when exposed to a low, medium, or high sound in everyday life, which does not cause similar reactions in a listener with normal hearing. The degree of reaction that the individual shows is closely related to the physical characteristics of the sound and not with the meaning of the sound or what kind of environment in which it is formed.^1^, ^2^, ^3^, ^4^, ^5^

Misophonia is defined as being able to tolerate loud sounds, while reacting to certain sound patterns even with low intensity or to certain sounds that occur in certain situations and conditions. The individual feels dislike, hatred, or antipathy for the sound. The degree of reaction is partially related to the physical characteristics of the sound and closely related to the meaning that the person attributes to that sound and their past experiences linked to it.^1^, ^2^, ^6^

Phonophobia, a subclass of misophonia, is the third DST. Phonophobia involves a reaction to certain sound patterns that occur under certain circumstances and conditions. Unlike misophonia, the feeling that predominantly accompanies phonophobia is fear. Individuals with phonophobia fear that normal environmental sounds will damage their ear, and therefore, they tend to avoid being in environments where there is a possibility of exposure to sounds.^7^, ^8^, ^9^

According to study results in the literature, since DST may occur in both individuals with normal hearing and individuals with hearing impairment, determining the pure-tone hearing threshold, loudness discomfort level, dynamic range, acoustic stapedius reflex test results do not provide consistent results about DST; hence, DST is not diagnosed at a sufficient level.^10^, ^11^, ^12^ Therefore, it is very important to obtain a detailed clinical history from the patient while determining the DST.^12^, ^13^ At this point, scales can help determine the severity of the problem and follow its course based on the patient’s history.

There are currently 4 scales that assess hyperacusis. The most commonly used one in English is the Hyperacusis Questionnaire (HQ)^14^ developed by Khalfa et al.; its Italian,^15^ Japanese,^16^ Dutch,^11^ and Turkish ^17^ versions are available. The second is the German Noise Sensitivity Scale (*Geräuschüberempfindlichkeit*) (GUF) developed by Nelting and Finlayson.^18^ The third is the Multiple-Activity Scale for Hyperacusis (MASH) developed by Dauman and Bouscau-Faure.^14^, ^19^ The fourth one is the Sound Tolerance Interview and Questionnaire Instrument (STIQI) developed by Sherlock and Formby in 2017.^20^ The number of scales evaluating hyperacusis in the world is limited. The Turkish validity and reliability study of HQ was not yet published at the time of this research, but it has been published very recently.^17^

There are three questionnaries that evaluate Misophonia. These are Misophonia Survey,^21^ Amsterdam Misophonia Questionnaire^22^ and MisoQuest.^23^ To the best of our knowledge, all of them has only English version. The Turkish validity and reliability study of Misofonia Survey still continues to be performed. No validated scale evaluating phonophobia has been published yet. There is no scale to be used to distinguish the subclasses of DST. Therefore, there is a need to develop a screening scale in Turkish to differentiate hyperacusis, misophonia, and phonophobia. Based on this information, the purpose of this study is to develop a scale that distinguishes DST's subclasses and to perform the validity and reliability study of the developed scale.

## Methods

This study, which was conducted as a cross-sectional field study, was approved by the local ethics committee (GO 17/998-19). The level of evidence rating of this study is three. Informed consent was obtained from all participants. Individuals from the general population who were over the age of 18 were asked: “Are environmental sounds disturbing for you?” Hearing screening was performed using *Oscilla* TSM 500 model audiometer at 1000–4000 Hz for those individuals who answered “Yes” to the abovementioned question and were willing to participate in the study.^24^ Failing for one or two ears in the hearing screening test, not giving consent, being under 18 and over 65 years of age were determined as exclusion criteria.

Out of 257 individuals in total who volunteered to participate in the study, 204 were included in the study group, and 53 were included in the control group. Individuals included in the study were provided a preliminary form with definitions of DST subclasses (hyperacusis, misophonia, phonophobia) and tinnitus, and also a patient demographic information form. In this form, participants were asked to mark which DST definitions were valid for them. Subsequently, DST type-specific evaluation forms, Patient Interview Form, and General Health Survey-12 form were provided to the participants.

Decreased Sound Tolerance Scale-Screening is an original screening scale developed by us to differentiate hyperacusis, misophonia, and phonophobia. At the beginning of the study, publications on DST in the literature were reviewed, experts working on the subject in the field were consulted, statements of patients about the complaints were considered, and the largest possible item pool was created. The items of the candidate scale were submitted to five experts for content validity. Content Validity Rates (CVR) and Content Validity Index (CVI) were calculated. Accordingly, experts evaluated each item using a 3-item Likert scale (1 = Required, 2 = Useful but not required, 3 = Not required). CVI value was calculated by finding the average of the total CVRs of the items to be included in the target scale. Pilot application was conducted with the remaining items. Subsequently, the study was initiated. The scale was applied in the form of reading and answering by individuals. Each item was scored between 0 and 3 (0 = Never; 3 = Always) according to the Likert system. As the score increased, the severity of the symptom increased. If the score for a specific subclass/subclasses was deemed high, it indicates that DST of the person is associated with that subclass.

All numerical, nominal, and ordinal data of the participants were uploaded to SPSS 23.0. Statistical analysis was performed using SPSS 23.0 and AMOS statistical programs. Descriptive statistics of the study were provided as frequency analysis for nominal and ordinal values and as mean and standard deviation for numerical values. Differences between groups were analyzed by testing the significance of the difference between two independent groups (*t*-test) and Chi-Square test. Item-total correlation and item differentiation analysis were implemented by independent sample *t*-test. Structure validity was analyzed by factor analysis [Explanatory Factor Analysis (EFA), Confirmatory Factor Analysis (CFA)]. Comparison of individuals with and without symptoms was made by Mann-Whitney *U* test. ROC analysis was performed for validity of the scale based on reference; accuracy rate, sensitivity, selectivity values, and cut-off points were provided. Internal consistency Cronbach’s alpha coefficient was determined for reliability analysis. Test-retest method was used to calculate intra-class correlation coefficients and Kappa coefficients.

## Results

It was predicted that the screening scale we aimed to develop would consist of approximately 20–25 items. Since there was no power analysis method for a newly developed scale, it was decided to reach 10 times the estimated number of items, which was a total of 250 participants.

Among 257 participants in total who volunteered to participate in the study and met the inclusion criteria, 204 were included in the study group and 53 were included in the control group. 189 of 257 subjects were female (73.5%), and 68 were male (26.5%). The mean age of the study group was 34.64 ± 9.88; the mean age was 33.66 ± 7.61 in the control group. 40.1% of the 204 participants in the study group had hyperacusis, 16.6% had phonophobia, and 81.8% had misophonia.

The items of the candidate scale, which consisted of 106 items in total, were submitted to five experts for content validity. Since the CVR value of items should be 0.99 for five experts,^25^ once the items with low CVRs were removed, 13 items remained in the hyperacusis section, 6 in the phonophobia section, and 14 in the misophonia section (Totally 33 items). The (CVI) was calculated as 1 by finding the average of the total CVRs of the items to be included in the target scale. Opinion was received that the questions in the measurement instrument were clearly intended for the information about the participants being investigated.

Subsequently, pilot application of the scale was conducted with the remaining 33 items. At this stage, it was decided that the sentence structures of the items were clear and understandable based on the information received from the participants for whom the scale was applied, thus face validity analysis was completed. There was no need to remove any item. It was decided that the actual study can start with the current state of the scale. The study was terminated when the number of targeted participants was reached.

It was decided to remove the items “H22. In order not to hear the noise, I use earplugs/protectors” of the hyperacusis section of DSTS-S and “F6. I can't go out without wearing an ear protector in my ears” of the phonophobia section, since they would not contribute to the scale as the item-total correlation coefficient was below 0.25.^26^ The total Cronbach alpha value of the hyperacusis section of the scale increased from 0.877 to 0.881 when item H22 was removed from the scale. Similarly, the total Cronbach alpha value of the phonophobia section increased from 0.758 to 0.775 when item F6 was removed from the scale. The total Cronbach alpha value of the Misophonia section was 0.938.

In order to assess item differentiation, the item averages were compared with the independent sample *t*-test for 27% groups that received the highest and lowest score from the total score of the scale. *P* < 0.01 was obtained for all items of hyperacusis, phonophobia, and misophonia sections.

Factorial validity of the phonophobia section was tested with EFA. It was determined to exhibit a single factor structure and F1, F3, F7, F11, and F15 were under one factor. This factor accounts for 53.49% of total variance with its structure. Factor loads of the phonophobia section are provided in Table 1.

**Table 1:** Factor loads of the phonophobia section.

Rotated component matrix of the phonophobia section	
	Factor load
**P1**	0.797
**P3**	0.677
**P7**	0.782
**P11**	0.668
**P15**	0.723

Extraction method: principal component analysis.

Factor analysis of the misophonia section was performed with EFA by applying Varimax rotation with Kaiser normalization. It was determined that the misophonia section consisted of 2 components. The items in Factor 1 (M6, M8, M13, M14, M16) are items that assess emotional complaints, and items in Factor 2 (M18, M20, M23, M25, M31, M32, M33, M34, M35) are items that assess functional/social complaints. The misophonia section accounts for 64% of the total variance with this 2-factor structure. Factor 1 accounts for 56.60% of this variance, and Factor 2 accounts for 8.12% of it. Factor loads of the misophonia section are provided in Table 2.

**Table 2:** Factor loads of the misophonia section.

Rotated component matrix of the misophonia section		
	Factor load	
	Factor 1	Factor 2
M6	0.298	0.765
M8	0.273	0.830
M13	0.468	0.657
M14	0.418	0.688
M16	0.200	0.778
M18	0.763	0.370
M20	0.630	0.480
M23	0.793	0.215
M25	0.806	0.230
M31	0.698	0.247
M32	0.681	0.454
M33	0.572	0.458
M34	0.707	0.316
M35	0.706	0.319

Extraction method: Principal component analysis.

Rotation method: Varimax rotation with kaiser normalization.

Although Varimax rotation with Kaiser normalization was performed, EFA did not yield the results we predicted, since the factor loads of some items of the hyperacusis section were very close to each other in two dimensions. CFA was used to assess whether this model established on a theoretical basis is compatible with the data set. It was noted that the hyperacusis section had a single factor structure. Accordingly, H1, H4, H8, H10, H11, H12, H23, H25, H27, H28, H35, and H44 were under a single factor.

**Figure 1:**
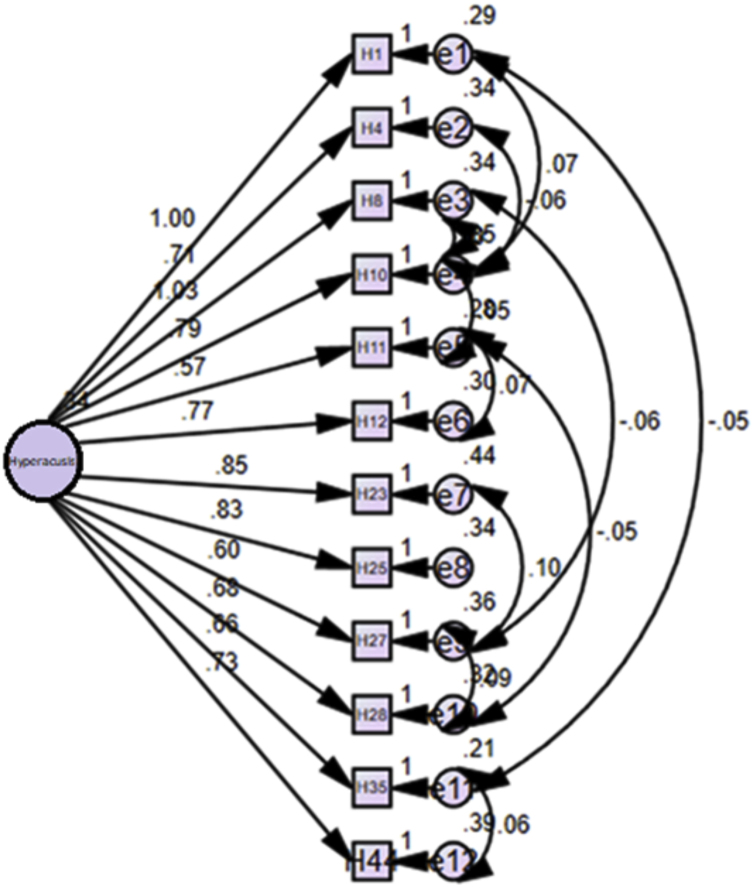
An analysis of goodness of fit with CFA for the hyperacusis section.

Based on the CFA result, Root Mean Square Error of Approximation (RMSEA) was 0.042, Degrees of Freedom (x²/df) was 1.432, Comparative Fit Index (CFI) was 0.982, Normed Fit Index (NFI) was 0.944, Non-Normed Fit Index/Trucker-Lewis Index (NNFI)/(TLI) was 0.972, and *p*-value was 0.033. RMSEA, x²/df, CFI and NFI index had good compatibility, and NNFI and *p*-value had acceptable compatibility. When all the values related to the model data compatibility of the hyperacusis section of the scale are examined, the established model had good compatibility to the data set, and therefore, it can be concluded that the hyperacusis section of the scale has structural validity. An analysis of goodness of fit with CFA for the hyperacusis section is provided in Fig. 1. The significance of the Goodness of Fit Statistics for the hyperacusis section of DSTS-S are provided in Table 3.

**Table 3:** The significance of the goodness of fit statistics for the hyperacusis section of DSTS-S.

Parameters	Values	Compatibility level
Root Mean Square Error of Approximation (RMSEA)	0.042	Good compatibility (0 ≤ RMSEA ≤0,05)
Degrees of Freedom (x²/df)	1.432	Good compatibility (0≤ x²/df ≤2)
Comparative Fit Index (CFI)	0.982	Good compatibility (>0.97)
Non-Normed Fit Index/Trucker-Lewis Index (NNFI)/(TLI)	0.972	Good compatibility (>0.97)
*p*-value	0.033	Acceptable compatibility (0.01 ≤ p ≤ 0.05)
Normed Fit Index (NFI)	0.944	Acceptable compatibility (>0.90)

In the examination of all descriptive statistics, the Mann-Whitney *U* test was used to test significant differences between 2 groups, as the total score data of all three groups did not show a normal distribution. There was a statistically significant difference between the median values of hyperacusis of participants with and without hyperacusis (*p* <  0.05). There was a statistically significant difference between the median values of phonophobia of participants with and without phonophobia (*p* <  0.05). There was a statistically significant difference between the median values of misophonias of participants with and without misophonia (*p* <  0.05). In addition, there was a statistically significant difference between the medium values of misophonia items Factor 1 and Factor 2 (*p* < 0.05). Median, interquarter interval, mean ± standard deviation, minimum value, and Maximum value data for HTSs, PTSs, and MTSs of participants with and without symptoms are presented in Table 4.

**Table 4:** Median, interquarter interval, Mean ± standard deviation, minimum value, and Maximum value data for HTSs, PTSs, and MTSs of participants with and without symptoms.

	Symptoms (+)					Symptoms (-)					
	Median	IQR (25–75)	Mean ± SD	Min.	Max.	Median	IQR (25–75)	Mean ± SD	Min.	Max.	*p*- value
Hyperacusis	14.0	8.0	13.5 ± 6.0	1.0	26.0	6.0	5.0	6.8 ± 4.3	0.0	24.0	0.000
Phonophobia	3.0	4.0	3.1 ± 2.6	0.0	9.0	0.0	1.0	0.6 ± 1.3	0.0	8.0	0.000
Misophonia											
Total	12.0	11.0	12.6 ± 8.2	0.0	38.0	2.0	7.0	4.9 ± 6.8	0.0	35.0	0.000
Factor 1	9.0	9.0	9.8 ± 6.0	0.0	27.0	2.0	6.0	3.8 ± 4.8	0.0	20.0	0.000
Factor 2	2.0	4.0	2.8 ± 2.7	0.0	13.0	0.0	1.2	1.1 ± 2.2	0.0	15.0	0.000

IQR (25–75), interquarter interval; mean ± SD, mean ± standard deviation; min., minimum value; max., maximum value.

**Figure 2:**
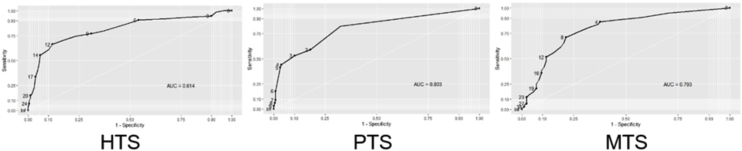
ROC curves for total scores.

Since the DSTS-S is a screening scale, no cut-off point was found in the ROC analysis that yielded sensitivity of 85%, for all three parts. The integer value closest to the specified sensitivity was selected as the cut-off point. The following was achieved for HTS: AUC = 0.814, 95% CI 0.749–0.878, *p* =  0.000. The following was achieved for PTS: AUC = 0.803, 95% CI 0.719–0.886, *p* =  0.000. The following was achieved for MTS: AUC = 0.793, 95% CI 0.731–0.855, *p* =  0.000. As a result of ROC analysis, it was found that HTS was useful in predicting the presence of hyperacusis, PTS was useful in predicting the presence of phonophobia, and MTS was useful in predicting the presence of misophonia. Cut-off point was calculated as ≥ 7 for HTS (Sensitivity: 0.864; Specificity: 0.517), ≥1 for PTS (Sensitivity: 0.823, Specificity: 0.675) and ≥ 4 for MTS (Sensitivity: 0.861; Specificity: 0.623). ROC curves for total scores are provided in Fig. 2.

Cronbach’s alpha was 0.881 for the hyperacusis section, 0.775 for the phonophobia section, and 0.938 for the misophonia section. Based on these values, hyperacusis and phonophobia sections had a good degree of reliability, and the misophonia section was perfectly reliable.

The intra-class correlation coefficient was 0.833 for the hyperacusis section, 0.752 for the phonophobia section, and 0.834 for the misophonia section. Accordingly, it was found that the phonophobia section had a very good level of reliability, and the hyperacusis and misophonia section exhibited a high level of reliability, so all three parts of the scale were stable.

Test-retest reliability of HTS, PTS, and MTS for the scale was analyzed by Kappa test, which is used in dependent data groups. Accordingly, *p* < 0.001 and Kappa numbers of test-re-test compatibility analysis of HTS, PTS, and MTS were 0.827 (perfect fit), 0.608 (moderate fit), and 0.526 (moderate fit), respectively. Accordingly, all three parts of the scale were consistent based on time.

Score range for each DST subclass for DSTS-S target scale is 0–36 for the 12-item Hyperacusis section, 0–15 for the 5-item Phonophobia section, and 0–42 for the 14-item Misophonia section. Among subclasses, the one with the highest total score indicates that it is the primary DST. The 31-item Decreased Sound Tolerance Scale-Screening (DSTS-S) is presented in Table 5.

**Table 5:** The 31 item Decreased Sound Tolerance Scale-Screening (DSTS-S). Explanation: The purpose of this scale is to help identify sound irritation. For each item, choose one among the applicable options of “never”, “sometimes”, “usually”, and “always”. Answer the questions on the basis of your experiences in the previous 7 days.

Hyperacusis	Never	Sometimes	Usually	Always
1. Loud noises irritate my ears.				
2. I am experiencing problems with my spouse/family due to my hypersensitivity to sound.				
3. Very loud noises make me angry.				
4. If there is a loud noise in the environment, I immediately go elsewhere.				
5. I don't enjoy music since I have become hypersensitive to sound.				
6. I avoid going to noisy environments, such as cinemas, concerts, fireworks show, restaurants, and bars because they cause pain/discomfort.				
7. I have difficulty following conversations in noisy environments.				
8. I am partially sensitive to street sounds and get uncomfortable.				
9. I have difficulty listening to announcements in noisy environments such as airports and train stations.				
10. I automatically cover my ears with my hands when I hear a slightly loud noise.				
11. Being irritated by noise makes me depressed.				
12. Being disturbed by noise prevents me from working.				
Total Score for Each Column				
Total points				

Phonophobia	Never	Sometimes	Usually	Always
13. I am afraid that some sounds, even if they are low-intensity, will damage my ears.				
14. I avoid going to noisy places such as cinemas and restaurants because I fear that it will hurt my ears.				
15. I am very afraid of sounds.				
16. I am afraid of some sounds that others are not.				
17. I'm afraid of going deaf because of loud noises.				
Total Score for Each Column				
Total points				

Misophonia: Answer by thinking about the sound of .................	Never	Sometimes	Usually	Always
18. Being disturbed by such sounds negatively affects my work life.				
19. Being disturbed by such sounds affects my whole life negatively.				
20. Being disturbed by such sounds affects my family relationships.				
21. Being disturbed by such sounds affects my ability to be around people.				
22. I worry that my whole life will be affected due to my irritation of such sounds.				
23. Such sounds make me nervous and irritable.				
24. I find it difficult to ignore such sounds in my daily life.				
25. I am disturbed by some sounds that others are not.				
26. I get angry at sounds that others are not bothered with.				
27. I want to punch the person making such sounds.				
28. I want to escape when I hear such sounds.				
29. I would like to cover my ears with my hands when I hear such sounds.				
30. Such sounds cause me to feel hatred and hold a grudge against the person (making the sound).				
31. Such sounds disgust me.				
Total Score for Each Column				
Total points				

Evaluation of DSTS-S:		
Disorders	Present	Not present
Hyperacusis	≥7	<7
Phonophobia	≥1	<1
Misophonia	≥ 4	<4

Thoughts/Comments:

## Discussion

At the beginning of the study, DSTS-S consisted of 106 items (49 for hyperacusis, 40 for misophonia, 17 for phonophobia). After the content validity analysis with experts, a total of 33 items remained including 13 from the hyperacusis section, 6 from the phonophobia section, and 14 from the misophonia section. At this stage, the experts provided an opinion that the questions in the measuring instrument were intended for the information on the subject being investigated.

Subsequently, pilot application of DSTS-S was conducted with 33 items. Based on the information received from the participants for whom the scale was applied, it was decided that the sentence structures of the items were understandable, clear, and appropriate for people from outside the field to understand; therefore, the validity analysis of the scale was also completed. Since there was no need to remove any item, it was decided to start the actual study for the scale in its current state.

According to the item-total correlation analysis of DSTS-S, H22 and F6 items had to be removed as they had no contribution to the scale. The reason why these two items were not useful can be explained by the fact that the use of ear protectors is hardly known in the Turkish society and (even in terms of occupational safety) is not a widespread practice. In potential validity and reliability studies of DSTS-S in other cultures, if item-total correlations of these two items are examined and determined that they contribute to the scale, it is proposed that they should be added as well. Item-total correlation coefficients and Cronbach alpha values of all three sections demonstrated that the hyperacusis section and the phonophobia section of DSTS-S had a good level of reliability, and the misophonia section had a perfect level of reliability. It was also established that the difference between the items was large; therefore, each item had a distinctive feature. The number of items in the final form of DSTS-S decreased to a total of 31 (12 in hyperacusis section, 5 in phonophobia section, 14 in misophonia section).

Factorial validity of DSTS-S was tested with EFA. It was determined that the phonophobia section consisted of a single component, and the misophonia section consisted of 2 components. Factorial validity of the hyperacusis section was analyzed by CFA, and it was determined to have a single-factor structure. When all the values related to the model data compatibility of the hyperacusis section of the scale were examined, the established model was adequately compatible with the data, and therefore, it can be concluded that the hyperacusis section of DSTS-S has structural validity.

Median value for HTSs of the group with hyperacusis was statistically significantly higher compared to the group without hyperacusis. Median value for PTSs of the group with phonophobia was statistically significantly higher compared to the group without phonophobia. Similarly, median value of MTSs of the group with misophonia was statistically significantly higher compared to the group without misophonia. Accordingly, it was found that all three parts of the scale were able to distinguish symptoms (+) individuals and symptoms (−) individuals.

Based on the ROC analysis, it was found that HTS from DSTS-S was useful in predicting the presence of hyperacusis, the PTS was useful in predicting the presence of phonophobia, and the MTS was useful in predicting the presence of misophonia. The cut-off point was ≥ 7, ≥ 1, and ≥ 4 in hyperacusis, phonophobia, and misophonia sections, respectively. Thus, compatibility validity of DSTS-S was achieved.

The reliability of DSTS-S was assessed by calculating internal consistency Cronbach's alpha coefficients; a good level of reliability was determined for the hyperacusis and phonophobia sections, and an excellent level of reliability was determined for the misophonia section. Test-retest reliability was assessed, and the obtained intra-class correlation coefficients showed high reliability for the hyperacusis section and the misophonia section and good reliability for the phonophobia section. Thus, it was determined that the scale showed stability.

It was determined that HTS had excellent compatibility in predicting the presence of hyperacusis. It was established that PTS had moderate and adequate compatibility in predicting the presence of phonophobia and MTS in predicting the presence of misophonia. Accordingly, it was determined that all three parts of DSTS-S showed no change based on time.

DSTS-S consists of 3 subgroups: hyperacusis, misophonia, and phonophobia. The hyperacusis section consists of 12 items that assess the functional/social and emotional complaints caused by the individual's hyperacusis; phonophobia section consists of 5 items; and the misophonia section consists of 14 items. The items in Factor 1 of the misophonia section assess emotional complaints, and the items in Factor 2 assess functional/social complaints.

Among DST subclasses, hyperacusis cases should be referred to an audiologist for treatment/therapy, while phonophobia and misophonia cases should be referred to a psychiatrist. By calculating the total score of items for subclasses of DST in DSTS-S, it is determined that the subclass with the higher score is the primary symptom. With this feature, DSTS-S guides the clinician/audiologist on which symptom should be focused and to which discipline the patient should be referred. This will prevent the patient from wasting time unnecessarily presenting to different clinics and the economic damage caused by unnecessary examinations.

## Conclusion

DSTS-S is easy to apply and takes a short time to answer (2.5–4 min) as it has a simple, clear, understandable sentence structure, low number of items, no open-ended questions, and having a Likert structure. DSTS-S, which has 31 items in total, is a scale with a proven compatibility in which each item correlates significantly with the scale and the items constitute a whole in themselves. As it is the first scale to evaluate DST subclasses together, it can be used in research related to DST.

### Limitations of the study

Include the absence of articles on phonophobia except for case studies, low number of phonophobia items, and lower number of participants with phonophobia complaints.

## Funding

This research did not receive any specific grant from funding agencies in the public, commercial, or not-for-profit sectors.

## Conflicts of interest

The authors declare no conflicts of interest.
